# Changes in Cell-Mediated Immunity (IFN-γ and Granzyme B) Following Influenza Vaccination

**DOI:** 10.3390/v13061137

**Published:** 2021-06-13

**Authors:** Naruhito Otani, Kazuhiko Nakajima, Kaori Ishikawa, Kaoru Ichiki, Takashi Ueda, Yoshio Takesue, Takuma Yamamoto, Susumu Tanimura, Masayuki Shima, Toshiomi Okuno

**Affiliations:** 1 Department of Public Health, Hyogo College of Medicine, Nishinomiya 663-8501, Hyogo, Japan; shima-m@hyo-med.ac.jp; 2 Department of Infection Control and Prevention, Hyogo College of Medicine, Nishinomiya 663-8501, Hyogo, Japan; nakajima@hyo-med.ac.jp (K.N.); i-kaori@hyo-med.ac.jp (K.I.); ichiki@hyo-med.ac.jp (K.I.); taka76@hyo-med.ac.jp (T.U.); takesuey@hyo-med.ac.jp (Y.T.); 3 Department of Legal Medicine, Hyogo College of Medicine, Nishinomiya 663-8501, Hyogo, Japan; tk-yamamoto@hyo-med.ac.jp; 4 Department of Public Health Nursing, Mie University Graduate School of Medicine, Tsu 514-0001, Mie, Japan; aruminat@gmail.com; 5 Department of Microbiology, Hyogo College of Medicine, Nishinomiya 663-8501, Hyogo, Japan; tmokuno@hyo-med.ac.jp

**Keywords:** cell-mediated immunity, granzyme B, influenza, IFN-γ, vaccine

## Abstract

Interferon gamma (IFN-γ) is considered a key moderator of cell-mediated immunity. However, little is known about its association with granzyme B, which plays an important role in the effector function of cytotoxic T lymphocytes (CTLs). In the present study, we collected blood samples from 32 healthy adults before and after vaccination with inactivated influenza vaccine in 2017/18 to measure the levels of IFN-γ and granzyme B, which play roles in cell-mediated immunity, and hemagglutination inhibition (HAI) antibody, which plays a role in humoral immunity. The levels of IFN-γ and granzyme B were significantly correlated both before and after vaccination. Furthermore, the post-vaccine fold increases in the IFN-γ and granzyme B levels were significantly correlated. The levels of IFN-γ and granzyme B decreased five months after vaccination in more than half of the subjects who exhibited an increase in IFN-γ and granzyme B at two weeks post-vaccination. This is the first study to investigate the correlation between IFN-γ and granzyme B levels following influenza vaccination. Our study suggests that both IFN-γ and granzyme B can be used as markers of cell-mediated immunity.

## 1. Introduction

In Japan, the inactivated quadrivalent influenza vaccine (IIV4) has been used since 2015/16. The vaccine used in the winter of 2017/18 targeted the following: A/Singapore/GP1908/2015 IVR-180 (H1N1) influenza A virus, A/Hong Kong/4801/2014 X-263 (H3N2) influenza A virus, B/Phuket/3073/2013 (Yamagata lineage) influenza B virus, and B/Texas/2/2013 (Victoria lineage) influenza B virus [1].

In the same season, the cases of influenza in Japan consisted of influenza A/H1N1 (23%), influenza A/H3N2 (32%), and influenza B (45%; 96% Yamagata lineage) [2,3]. This was the second year since 2004/05 in which influenza B was the most prevalent. For all types, the incidence was most common in children under the age of 15 [3].

According to a report published by the Centers for Disease Control and Prevention (CDC), influenza A and B accounted for 67.6% and 32.4% of the total U.S. cases in 2017/18, respectively. Specifically, influenza A/H1N1 accounted for 84.9% of all influenza A cases, and the Yamagata lineage accounted for 88.8% of all influenza B cases [4]. The efficacy of the influenza vaccination was reported to be 62%, 22%, and 48% for influenza A/H1N1, influenza A/H3N2, and influenza B/Yamagata lineage, respectively [5].

In addition to humoral immunity, cell-mediated immunity is thought to play an important role in immunity against influenza [6]. Interferon gamma (IFN-γ) is an important cytokine secreted by Type 1 T helper (Th1) cells, and it plays an important role in cell-mediated immunity. Thus, in previous studies we examined IFN-γ levels to assess the effect of influenza vaccination on cell-mediated immunity [7,8,9]. Both CD4+ and CD8+ T cells are considered important in building immunity against influenza, and IFN-γ was demonstrated to help induce CD8+ T cells. Among CD8+ T cells, CD8+ cytotoxic T lymphocytes (CTLs) are known to mediate viral clearance and recovery from infection. Granzyme B is an important effector molecule produced by CTLs [10], and previous studies have investigated its role in the context of vaccination [11,12]. However, little is known about the relationship between IFN-γ and granzyme B. In the present study, we evaluate the effect of the inactivated influenza vaccine on cell-mediated immunity by quantifying the IFN-γ and granzyme B levels before and after vaccination. Moreover, we determine whether the levels of both molecules could be used to evaluate the efficacy of influenza vaccines in inducing cell-mediated immunity.

## 2. Materials and Methods

### 2.1. Study Population and Vaccine

A total of 32 healthy adults (age: 33–59 years; 14 male and 18 female) were registered for the study. All subjects received the influenza vaccine between September and December 2017. One dose (0.5 mL) of the IIV4 (BIKEN, Osaka, Japan, LOT HA173B) vaccine was administered subcutaneously. Blood was collected before vaccination and two weeks and five months after vaccination to measure cell-mediated (IFN-γ and granzyme B levels) and humoral (hemagglutination inhibition (HAI) antibody titers) immunity. The study was approved by the Research Ethics Board of the Hyogo College of Medicine.

### 2.2. Antigens

The following strains from the IIV4 vaccine for 2017/18 were used: A/Singapore/GP1908/2015 IVR-180 (H1N1), A/HongKong/4801/2014 X-263 (H3N2), B/Phuket/3073/2013 (Yamagata lineage), and B/Texas/2/2013 (Victoria lineage). These antigens were provided by BIKEN.

### 2.3. IFN-γ and Granzyme B Assays

The assays were performed as previously described for IFN-γ [7]. Briefly, heparinized whole blood (100 μL) was added to flat-bottom microtiter plates and incubated with varying amounts of influenza antigens (HA titer: 10 μg/mL) diluted in RPMI 1640 medium in a final volume of 200 μL/well. The incubations were conducted within 1 h of drawing the blood samples. The supernatants (100 μL) were collected after 48 h of incubation and stored at −80 °C until further use. IFN-γ and granzyme B concentrations were measured using enzyme-linked immunosorbent assays (IFN-γ Assay Kit (ebioscience, San Diego, CA, USA) and a Human Granzyme B ELISA development kit (MABTECH, Nacka Strand, Sweden), according to the manufacturer’s instructions. Phytohemagglutinin (final concentration: 2.5 μg/mL) and medium were used as positive and negative controls, respectively. The amount of IFN-γ released in the negative control wells was < 4 pg/mL in all experiments.

Subjects with a ≥1.5-fold increase in IFN-γ and granzyme B production were considered to have positive reactions because, in our previous study, the fold increase in the IFN-γ production of influenza-infected subjects in response to vaccination was not higher than 1.5 [7].

### 2.4. Antibody Titration

HAI antibody titers were measured using the vaccine strains. Each sample was diluted 1:10 with receptor-destroying enzyme and the serum HAI titer was measured using an influenza virus HAI test (Denka Seiken Co., Tokyo, Japan). The final dilution factor that inhibited hemagglutination was considered to be the HAI titer. HAI titers ≥ 1:10 were considered positive and those < 1:10 were considered negative. The HAI titer was measured by a commercial laboratory (SRL, Inc., Tokyo, Japan).

### 2.5. Statistical Analyses

To determine the correlation between two sets of immunologic test results, Spearman rank correlations were calculated, followed by tests of no correlation with a significance level of *p* < 0.05. All statistical analyses were performed using SPSS (version 22; IBM Co., Armonk, NY, USA).

## 3. Results

### 3.1. Changes in Cell-Mediated Immunity (IFN-γ, Granzyme B) after Influenza Vaccination

**Figure 1:**
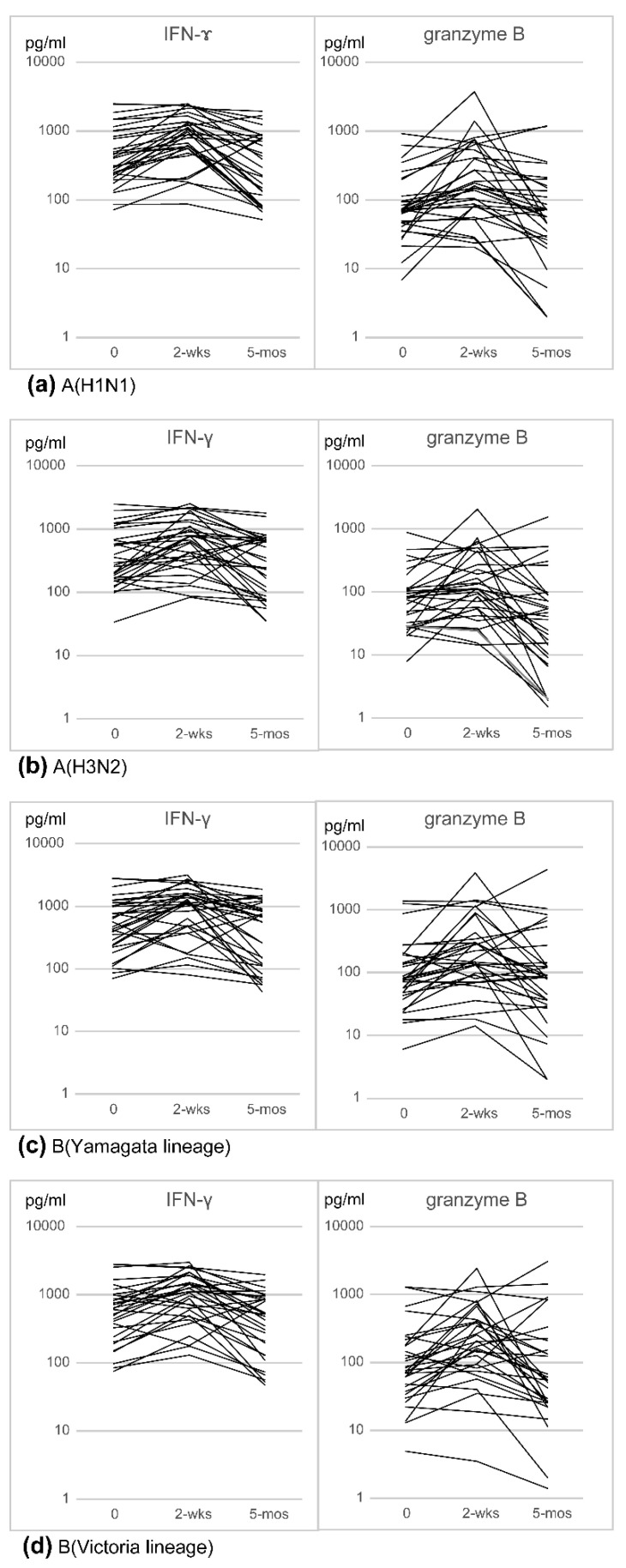
Cell-mediated immunity (IFN-γ and granzyme B levels) to the four vaccine antigens: (**a**) A(H1N1); (**b**) A(H3N2); (**c**) B(Yamagata lineage); (**d**) B(Victoria lineage). IFN-γ and granzyme B levels were measured at pre-vaccination (0), two weeks post-vaccination (2 wks), and five months post-vaccination (5 mos).

IFN-γ and granzyme B production in response to the A(H1N1), A(H3N2), B(Yamagata lineage), and B(Victoria lineage) antigens was examined before vaccination, and two weeks and five months after vaccination. In more than half of the subjects, the IFN-γ and granzyme B levels increased two weeks after vaccination and subsequently decreased (Figure 1). The geometric mean concentrations (GMCs) of IFN-γ and granzyme B increased two weeks after vaccination and subsequently decreased (Table 1). Furthermore, the percentage of subjects with a ≥1.5-fold increase in IFN-γ and granzyme B levels two weeks after vaccination was approximately 50%. However, the percentage was lower than 50% five months after vaccination (Table 1).

### 3.2. Correlation between IFN-γ and Granzyme B

The correlation between IFN-γ and granzyme B levels before and after vaccination was examined using Spearman’s correlation coefficient (Table 2). The magnitude of change from the pre- to post-vaccine level was correlated for all antigens. Similarly, the rate of change from pre- to post-vaccine level was correlated for all antigens. This suggests that the IFN-γ and granzyme B responses to influenza vaccines are similar. However, there was no significant correlation between cell-mediated immunity (IFN-γ and granzyme B levels) and antibody titer.

### 3.3. Changes in Antibody Titers after Vaccination

The European Agency for the Evaluation of Medicinal Products (EMEA) guidelines indicate that, for individuals aged 18–60 years, vaccine efficacy should be evaluated based on pre- and post-vaccine HI titers and that vaccines should meet at least one of the following criteria: (1) post-vaccine HAI titer of ≥1:40 in at least 70% of individuals; (2) pre-vaccine HAI titer of <1:10 and post-vaccine HAI titer of ≥1:40, or over 4-fold increase in HAI titer in at least 40% of individuals; (3) GMT ratio of >2.5 [12]. Based on the guidelines, these criteria were evaluated:

(a) Percentage of subjects with an HAI titer of ≥1:40.

As shown in Table 3, a post-vaccine HAI titer of ≥1:40 in at least 70% of individuals was only found for A(H3N2) two weeks after vaccination (90.6%, 29/32). However, the percentage was also close to 70% at pre-vaccination (68.8%, 22/32) and returned to the baseline five months after vaccination.

(b) Percentage of subjects with a pre-vaccine HAI titer of <1:10 and post-vaccine HAI titer of ≥1:40, or over 4-fold increase in HAI titer.

As shown in Table 3, the highest percentage of positive subjects according to this criterium was found for A(H1N1) two weeks after vaccination. However, the percentage of positive subjects was always <40%, regardless of the antigen tested.

(c) GMT ratio between the post-vaccination and pre-vaccination HAI GMT titers.

As shown in Table 3, a GMT ratio > 2.5 was only found for A(H1N1) both two weeks and five months after vaccination.

## 4. Discussion

Previous studies have suggested that inactivated vaccines only trigger humoral immunity. However, recent studies have suggested that inactivated vaccines also elicit a certain level of cell-mediated immunity. Specifically, cell-mediated immunity is thought to play an important role in protecting against influenza [13]. The role of IFN-γ in cell-mediated immunity has been well-characterized in several studies using different methods [14,15,16]. In general, these studies have examined cell-mediated immunity by quantifying the production of IFN-γ by monocytes isolated from blood in response to antigens. We have previously developed an assay that facilitates the process by using whole blood [7] and demonstrated its suitability for the evaluation of cell-mediated immunity [9]. Evaluation by cell-mediated immunity has been put to practical use in *Mycobacterium tuberculosis* (TB). Interferon Gamma Release Assays (IGRAs) are used as a method for evaluating cell-mediated immunity. In Japan, IGRAs have been used for applications such as contact investigations and the TB screening of healthcare workers [17]. Cell-mediated immunity is also thought to be important in the development of herpes zoster [18], and several methods focusing on cell-mediated immunity have been developed for herpes zoster [19,20].

Although the role of IFN-γ and granzyme B in cell-mediated immunity in the context of vaccination has been explored, the relationship between these two factors remains unknown. In the present study, we used our method to quantify IFN-γ and granzyme B production in whole blood from influenza-vaccinated individuals in response to influenza antigens. Then, we examined the correlation between the IFN-γ and granzyme B levels and found a significant correlation between the two, revealing that IFN-γ and granzyme B responses to the influenza vaccine occur in a similar manner. These results suggest that both IFN-γ and granzyme B can be examined to evaluate the efficacy of influenza vaccines in eliciting cell-mediated immunity.

Some studies have reported that IFN-γ and granzyme B are induced after vaccination [11,12,21]. However, this is the first study to show a correlation between IFN-γ and granzyme B levels following vaccination. This may be related to the fact that other methods require processing such as cell separation, making it difficult to rapidly assess cell-mediated immunity in many specimens. Our method was based on whole blood, and hence specimen handling was easier in our method than in other methods. Therefore, we believe that our method is relevant and can be applied in clinical settings. However, various types of vaccines are currently being used, and each vaccine needs to be examined in terms of the differences in cell-mediated immunity achieved.

The GMCFR ratio five months after vaccination was lower than that before vaccination. However, by excluding samples with a pre-vaccine IFN-γ level of over 1000 pg/mL, the GMCFR became closer to one. This suggests that this method should be tested in individuals with pre-vaccine IFN-γ levels of less than 1000 pg/mL. In future studies, the antigen concentrations for whole blood stimulation may need to be reduced.

The EMEA guidelines [22] were used to evaluate antibody titers. The criterium of a post-vaccine HAI titer of ≥1:40 in at least 70% of individuals was only met for A(H3N2); however, the percentage of subjects meeting the criterium was also close to 70% pre-vaccination (68.8%, Table 3).

As shown in Table 3, the greatest percentage of subjects with a pre-vaccine HAI titer of <1:10 and post-vaccine HAI titer of ≥1:40, or an over 4-fold increase in HAI titer, was found for A(H1N1) (12/32, 37.5%). This suggests that A(H1N1) is the most reacting vaccine antigen.

An increase in the GMT ratio > 2.5 after vaccination was only found for A(H1N1) (Table 3). However, the GMTs for A(H1N1), B(Yamagata lineage), and B(Victoria lineage) were similar to the pre-vaccine baseline five months after vaccination. Thus, it may be difficult to evaluate vaccine efficacy based on humoral immunity alone.

There are several limitations to our study. First, since none of the subjects were exposed to influenza virus, we were unable to examine the level of granzyme B in response to influenza infection. If the immune response to influenza infection is similar to the immune response to the vaccine, IFN-γ and granzyme B could serve as important additional indicators for evaluating the effectiveness of the vaccine. Second, although our method for cell-mediated immunity quantification is relatively easy to perform compared with conventional techniques, it requires that the blood samples be treated rapidly after collection. Thus, compared with methods for humoral immunity determination, our method may not be optimal for large-scale processing. Third, the antigens included in influenza vaccines change every year. In the future, it will be necessary to evaluate whether the changes in antigens affect the cell-mediated immune response.

In conclusion, we assessed the immune response following vaccination. We subsequently demonstrated that granzyme B and IFN-γ levels are positively correlated. Moreover, our study suggests that both IFN-γ and granzyme B can be examined to evaluate the efficacy of influenza vaccines in eliciting cell-mediated immunity.

## Figures and Tables

**Table 1:** Geometric mean concentrations and fold rises in the levels of antigen-specific IFN-γ and granzyme B in vaccinated subjects before and after vaccination, and percentage of subjects with ≥1.5-fold increase in IFN-γ and granzyme B levels after vaccination: (a) GMCs and GMCFRs upon the stimulation of whole blood with vaccine antigens; (b) percentage of subjects with ≥1.5-fold increase in IFN-γ and granzyme B levels upon vaccination.

(a)										
***n* = 32**	**IFN-** **γ** **: GMC (GMCFR)**					**Granzyme B: GMC (GMCFR)**				
	**Pre-vaccine**		**2 wks**	**5 mos**		**Pre-vaccine**		**2 wks**		**5 mos**
H1N1	429.9		801.1 (1.9)	307.5 (0.72)		76.3		173.2 (2.3)		53.9 (0.71)
H3N2	344.8		597.4 (1.7)	218.5 (0.6)		68.9		122.7 (1.8)		34.4 (0.5)
Yamagata lineage	490		835.1 (1.7)	349 (0.7)		87		196.8 (2.3)		79.3 (0.9)
Victoria lineage	531.5		909.7 (1.7)	350 (0.66)		87.7		187 (2.1)		66 (0.75)
(b)										
		**IFN-** **γ**					**Granzyme B**			
***n* = 32**		**2 wks**			**5 mos**		**2 wks**		**5 mos**	
H1N1		50% (16/32)			25% (8/32)		56.3% (18/32)		28% (9/32)	
H3N2		50% (16/32)			22% (7/32)		43.8% (14/32)		16% (5/32)	
Yamagata lineage		50% (16/32)			22% (7/32)		62.5% (20/32)		28% (9/32)	
Victoria lineage		62.5% (20/32)			19% (6/32)		65.6% (21/32)		28% (9/32)	

GMC—geometric mean concentration; GMCFR—geometric mean fold rise; 2 wks—two weeks post-vaccination; 5 mos—five months post-vaccination. 2 wks—two weeks post-vaccination; 5 mos—five months post-vaccination.

**Table 2:** Spearman’s correlation coefficients assessing the relationship between IFN-γ and granzyme B levels before and after vaccination.

*n* = 32	IFN-γ and Granzyme B			
	Pre-Vaccine	2 wks Post-Vaccine	5 mos Post-Vaccine	2 wks Post-Vaccine/Pre-Vaccine
H1N1	0.66 **	0.74 **	0.50 **	0.59 **
H3N2	0.62 **	0.71 **	0.60 **	0.56 **
Yamagata lineage	0.74 **	0.80 **	0.77 **	0.47 **
Victoria lineage	0.75 **	0.75 **	0.63 **	0.61 **

Notes: ** *p* < 0.01.

**Table 3:** Evaluation based on the criteria of the EMA guidelines: (a) percentage of subjects with HAI titers of ≥1:40; (b) percentage of subjects with HAI titers of <1:10 before vaccination and ≥1:40 after vaccination, or with HAI titers of ≥1:10 before vaccination and an increase of at least 4-fold after vaccination; (c) GMT ratios between post-vaccination GMTs and pre-vaccination GMTs.

(a)								
		**Percentage of Subjects with HAI ≥ 1:40**						
***n* = 32**		**Pre-Vaccine**			**2 wks**			**5 mos**
H1N1		15.6% (5/32)			56.3% (18/32)			43.8% (14/32)
H3N2		68.8% (22/32)			90.6% (29/32)			68.8% (22/32)
Yamagata lineage		31.3% (10/32)			53.1% (17/32)			40.6% (13/32)
Victoria lineage		18.8% (6/32)			34.4% (11/32)			31.3% (10/32)
(b)								
			**HAI**					
***n* = 32**			**2 wks**				**5 mos**	
H1N1			37.5% (12/32)				21.9% (7/32)	
H3N2			28.1% (9/32)				12.5% (4/32)	
Yamagata lineage			15.6% (5/32)				9.4% (3/32)	
Victoria lineage			15.6% (5/32)				12.5% (4/32)	
(c)								
***n* = 32**	**GMT (GMT Ratio)**							
	**Pre-Vaccine**			**2 wks Post-Vaccine**		**5 mos Post-Vaccine**		
H1N1	10.9			28.9 (2.7)		27.1 (2.5)		
H3N2	30.2			54.2 (1.8)		43.6 (1.4)		
Yamagata lineage	17.9			24.8 (1.4)		22.8 (1.3)		
Victoria lineage	12.2			17.2 (1.4)		17.9 (1.5)		

2 wks—two weeks post-vaccination; 5 mos—five months post-vaccination. 2 wks—two weeks post-vaccination; 5 mos—five months post-vaccination. Notes: GMT ratio requirements ≥ 2.5.

## Data Availability

All data generated and analyzed during this study are included in this article.
